# Seropositive for hepatitis B and C viruses is associated with the risk of decreased bone mineral density in adults: An analysis of studies from the NHANES database

**DOI:** 10.3389/fmed.2023.1120083

**Published:** 2023-03-22

**Authors:** Jiasheng Tao, Zijian Yan, Wenmian Huang, Tao Feng

**Affiliations:** ^1^The First Clinical Medical College, Guangzhou University of Chinese Medicine, Guangzhou, Guangdong Province, China; ^2^Affiliated Stomatological Hospital, Guangzhou Medical University, Guangzhou, Guangdong Province, China; ^3^Department of Orthopedics, Nantong Hospital of Traditional Chinese Medicine, Nantong, Jiangsu, China

**Keywords:** bone mineral density, NHANES, hepatitis, hepatitis B surface antigen, hepatitis C RNA

## Abstract

**Background:**

Some studies had reported that patients with viral hepatitis are at increased risk of reduced bone mineral density and even osteoporosis. However, the interaction between reduced bone mineral density (BMD) and viral hepatitis remains inconclusive. Therefore, our study collected hepatitis test results and bone mineral density from respondents in the NHANES database. The aim of this study was to investigate whether there is an association between hepatitis and a decrease in bone mineral density.

**Methods:**

The respondents with both hepatitis- and BMD-related indicators from the NHANES database in the United States from 2005–2010, 2013–2014, to 2017–2020 were collected for this study. BMD were compared between respondents who were positive and negative for respondents related to hepatitis B and C. BMD was measured using dual-energy X-ray absorptiometry of the femur and lumbar spine. Finally, multiple regression analysis was performed between hepatitis B surface antigen (HBsAg) and hepatitis C RNA (HCV-RNA) and BMD in the respondents.

**Results:**

A total of 15,642 respondents were included in the hepatitis B surface antigen-related survey. Of these, 1,217 respondents were positive for hepatitis B surface antigen. A total of 5111 hepatitis C RNA-related responders were included. Hepatitis C RNA-positive had 268 respondents. According to the results of the multiple regression analysis, the femoral BMD was significantly lower in HBsAg (+) respondents compared to HBsAg (–) respondents: −0.018 (−0.026, −0.009) (*P* < 0.01). Moreover, spinal BMD was significantly lower in HBsAg (+) respondents compared to HBsAg (–) respondents: −0.020 (−0.030, −0.010) (*P* < 0.01). According to the results of multiple regression analysis for hepatitis C RNA, HCV-RNA (+) respondents had significantly lower BMD compared to HCV-RNA (–) respondents: −0.043 (−0.059, −0.026) (*P* < 0.01).

**Conclusion:**

During the analysis of respondents in the NHANES database in the United States, positive tests for hepatitis B surface antigen and hepatitis C RNA were found to be associated with a reduction in BMD. Positive serology for these hepatitis indicators may increase the risk of reduced BMD. Of course, this conclusion still needs to be further confirmed by more large clinical trials.

## Introduction

Osteoporosis is caused by bone loss, which is a global public health problem that can easily lead to fractures with serious consequences and even death (1–3). Osteoporosis affects ~200 million people all over the world, with a prevalence of ~18.3% (4). Some studies have shown that patients with hepatitis are prone to reduced BMD, which, in turn, can increase the risk of osteoporosis. Moreover, osteoporotic fractures in patients with chronic hepatitis are on the rise worldwide (5–7).

According to some current reports, osteoporosis is one of the complications of hepatitis, and the prevalence of osteoporosis may be higher in patients with liver cirrhosis (8–10). About the effect of chronic hepatitis and cirrhosis on bone mineral density, some studies had pointed out that under the influence of various chronic inflammatory factors. This series of changes gradually leads to a loss of bone mass and a decrease in bone mineral density, which, in turn, increases the risk of osteoporosis (6, 11, 12).

Hepatitis virus infection is the most common pathogenic route of hepatitis. Viruses that cause viral hepatitis commonly include hepatitis A, B, C, D, and E viruses, and viral hepatitis is often an important cause of liver cirrhosis (13–15). There is still no clear consensus on the relationship between hepatitis and the reduction in BMD. Many studies had shown that hepatitis can lead to a decrease in BMD, and a number of studies had shown a weak association between the two. For these reasons, we compared BMD levels between positive and negative respondents for hepatitis virus-related indicators from the NHANES database in the United States to analyze the relationship between the two.

## Methods

### Study design and population

The data of our current study were obtained from the National Health and Nutrition Examination Survey (NHANES) for the period of 1999 to 2020. This database is a nationally representative survey of the civilian, de-institutionalization population of the United States conducted by the National Center for Health Statistics (NCHS) of the Centers for Disease Control and Prevention (CDC) (16). The data in the NHANES database contain five sections, such asdemographic data, dietary data, examination data, laboratory data, and questionnaire data. Informed consent was available for the content of all respondents in the NHANES database. The disclosure of this content has been approved by the NCHS Research Ethics Review Committee (17).

**Figure 1:**
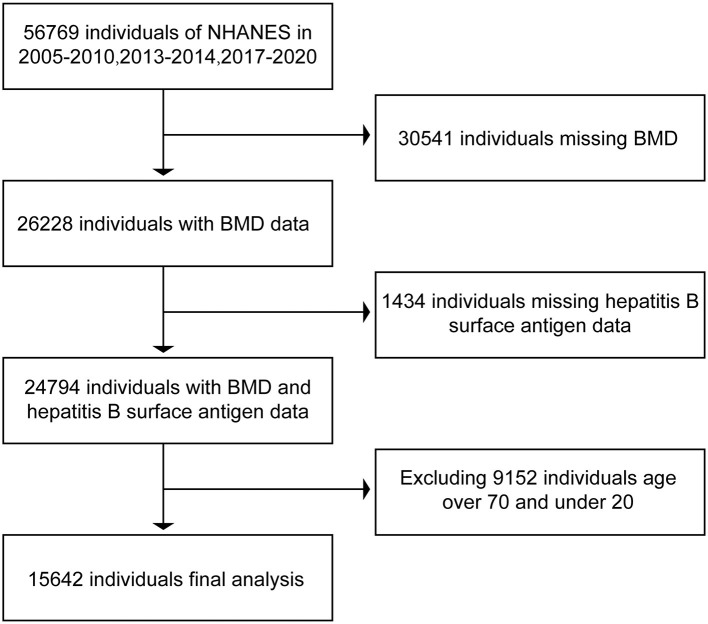
Flow chart of inclusion and exclusion of respondents associated with hepatitis B surface antigen.

**Figure 2:**
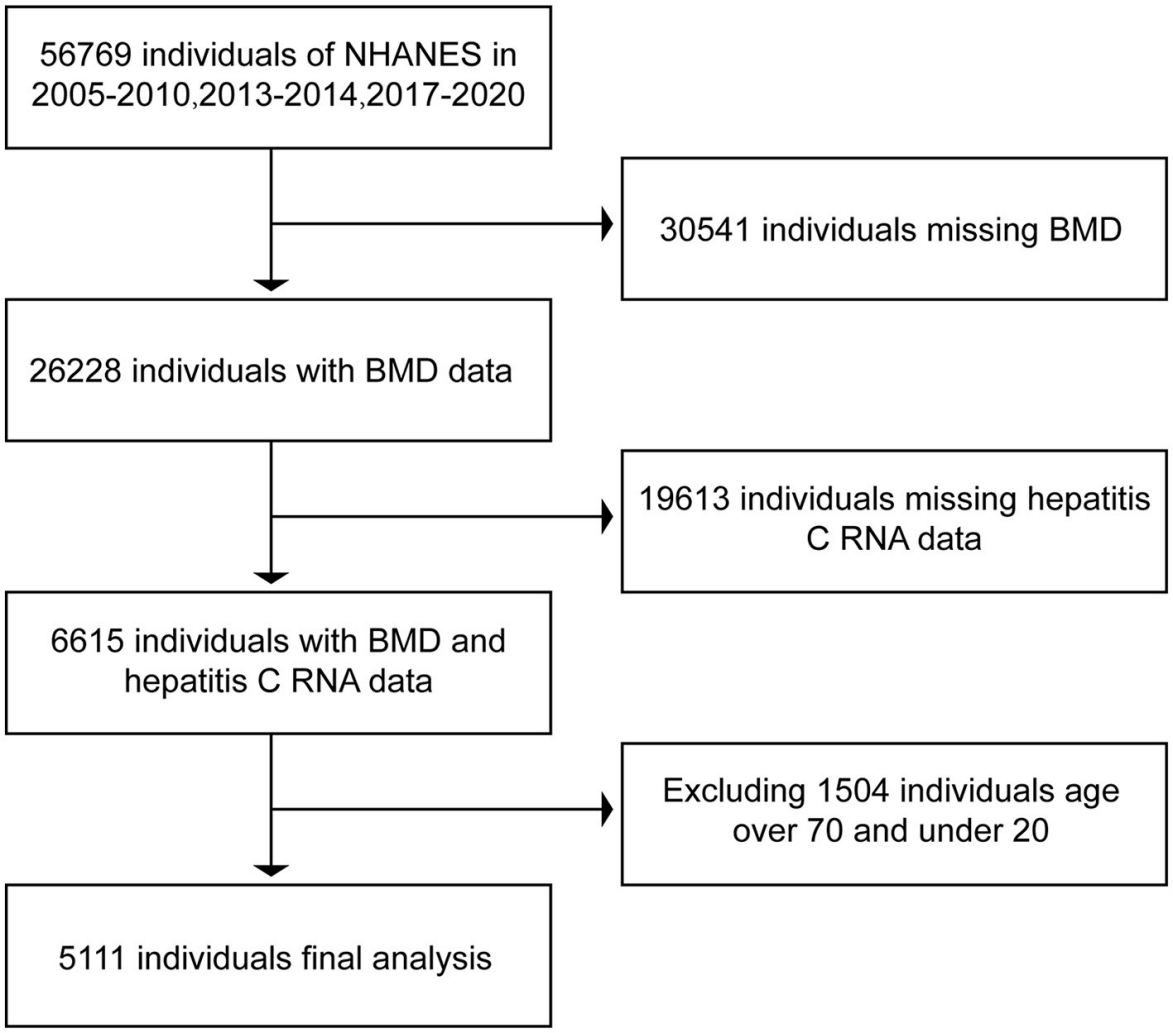
Flow chart of inclusion and exclusion of respondents associated with hepatitis C RNA.

In the NHANES database, we downloaded the data related to hepatitis B surface antigen, hepatitis C RNA, and BMD in respondents during the period of 2005–2010, 2013–2014, and 2017–2020. In bone imaging, there is interference in imaging, as the bones are still underdeveloped below the age of 20 years. In contrast, respondents older than 70 years of age enter the stage of senile osteoporosis and it is more prone to bone loss (18, 19), and the possible presence of senile osteoporosis can have an impact on the final results. Therefore, we selected data from respondents aged 20 years or older and younger than 70 years. After excluding data from non-compliant respondents, we ended up collecting 15,642 respondents with both hepatitis B surface antigen and BMD and 5,111 respondents with both hepatitis C RNA and BMD. The data were then collated and analyzed using R and Empower software. The BMD of serologically positive and serologically negative respondents for hepatitis B surface antigen and serologically positive and serologically negative respondents for hepatitis C RNA were compared separately to see if there were differences in BMD between positive and negative respondents. The specific inclusion and exclusion processes are shown in Figures 1, 2.

### Bone mineral density levels

Bone mineral density (BMD) is measured by using a dual X-ray absorptiometry (DXA) examination. Dual-energy X-ray absorptiometry (DXA) is the most widely accepted method of measuring bone mineral density due in part to its speed, ease of use, and low radiation exposure. DXA scans of the proximal femur were administered in the NHANES mobile examination center (MEC) from 2005–2010, 2013–2014, to 2017–March 2020 (20, 21).

### Hepatitis serology levels

Hepatitis B surface antigen is tested by using the VITROS HBsAg test, the VITROS HBsAg kit on the VITROS ECi/ECiQ Immunodiagnostic System and VITROS 3600 Immunodiagnostic System, and the VITROS Immunodiagnostic Product HBsAg Calibrator (22). Hepatitis C ribonucleic acid is tested by using the COBAS Amplicon HCV Monitor test. The COBAS Amplicon HCV Monitor version 2 0 (v2.0) is an *in vitro* nucleic acid amplification test for the quantification of hepatitis C virus RNA in human serum or plasma on the COBAS Amplicon analyzer (23).

### Assessment of covariates

In the NHANES database, there is a column for demographic data. In this column, we collected information on the age, gender, race, income level, education level, and other relevant information of the respondents. The race is categorized as Mexican American, other Hispanic, non-Hispanic White, non-Hispanic Black, and other. The household income levels are categorized as low income, middle income, and high income; education levels are categorized as below high school, high school or equivalent, college or above, and other.

In the dietary data column, information on the diet of the respondents is recorded, and it is possible to know about the nutritional intake of the respondents. In this column, we collected information from respondents about their intake of calcium and alcohol. The examination information column contains the data on the physical examination of the respondents. In this column, we collected not only the BMD data of the respondents but also the data of the respondents' body mass index (BMI).

In the column of laboratory test data, the results of the relevance of the respondents' tests are recorded. Here, we collected the data on the hepatitis B surface antigen and hepatitis C RNA of respondents. In addition, data on liver function, HDL, uric acid, creatinine, calcium levels, and blood glucose, which are all relevant covariates that may affect the results, were also collected in this column.

Finally, we collected data about smoking and the presence of diabetes in the respondents in the questionnaire column. Respondents were considered to have a smoking habit if they had smoked more than 100 cigarettes previously.

### Statistical analyses

For statistical analysis of the data, we used the R language 3.4.3 and EmpowerStats 2.0. According to the hepatitis indicators, the respondents who were positive and negative for hepatitis were divided into two different groups. In terms of data statistics, the data of categorical variables are expressed as numbers with percentages (*N*%), while the data of continuous variables are expressed as mean values with standard deviations (mean ± SD). Hepatitis as the exposure variable is a categorical variable, and BMD as the outcome indicator is a continuous variable. Therefore, the χ^2^ test (categorical variable) and the linear regression model (continuous variable) were used to calculate the difference in BMD among different groups. For analyzing the differences in BMD between the groups, three different multiple regression equation models were used. No adjustment was made for Model 1; Model 2 was adjusted for age, race, and gender; and Model 3 was adjusted for age, race, gender, income level, education level, BMI, smoking, alcohol consumption, calcium intake, HDL, uric acid, creatinine, blood calcium levels, blood glucose, and diabetes.

For the relationship between serological indicators of viral hepatitis and BMD, we used multiple regression model analysis, using a smooth curve fitting. For smooth curve fitting, the variables of adjustment were similar to that of Model 3. In addition, we performed stratified analyses, according to different ages and BMI. The age was divided into three groups: < 40, < 60, and ≥60 years old. The BMI was divided into four groups: < 18.5, 18.5–24.9, 24.9–30, and >30 kg/m^2^. A *P*-value of < 0.05 is considered statistically significant.

## Results

### Baseline characteristics of study participants

In this study, we initially collected relevant data from 56,769 respondents. A total of 31,975 respondents were excluded due to a lack of data related to hepatitis B surface antigen or bone mineral density. Moreover, 9,152 respondents older than 70 years of age and younger than 20 years of age were excluded. A total of 15,642 respondents were included in the final study. Of the 15,642 respondents included in the analysis, 1,217 were positive for hepatitis B surface antigen and the rest of the respondents are negative. Data related to hepatitis C RNA included a total of 56,769 respondents at first. Of these, 50,154 respondents were excluded due to lack of hepatitis C RNA or bone mineral density-related data, 1,504 respondents older than 70 years of age and younger than 20 years of age were excluded, and a total of 5,111 respondents were included in the study finally. A total of 5,111 respondents had hepatitis C RNA data, of which 268 were positive, and the rest were negative. The data relating to the included respondents are shown in Table 1.

**Table 1:** Baseline characteristics of respondents with BMD according to HBsAg or HCV-RNA seropositivity.

	**Hepatitis B and C related indicators**					
	**HBsAg (–)**	**HBsAg (**+**)**	***P*** **-value**	**HCV-RNA (–)**	**HCV-RNA (**+**)**	***P*** **-value**
*N*	14,425	1,217		4,843	268	
Age	47.94 ± 14.06	54.51 ± 10.94	< 0.01	56.87 ± 8.17	52.64 ± 9.32	< 0.01
Gender			< 0.01			< 0.01
Male (%)	7,276 (50.44%)	699 (57.44%)		2,415 (49.87%)	182 (67.91%)	
Female (%)	7,149 (49.56%)	518 (42.56%)		2,428 (50.13%)	86 (32.09%)	
AST	25.38 ± 15.33	29.90 ± 32.14	0.02	23.56 ± 13.34	58.39 ± 41.99	< 0.01
ALT	25.84 ± 19.19	29.02 ± 29.50	< 0.01	23.65 ± 18.31	61.68 ± 47.92	< 0.01
Creatinine	78.67 ± 37.26	81.41 ± 39.86	0.01	80.46 ± 38.60	88.12 ± 72.71	0.40
Blood.calcium	9.43 ± 0.37	9.41 ± 0.37	0.24	9.36 ± 0.36	9.39 ± 0.39	0.01
Uric.acid	5.39 ± 1.39	5.58 ± 1.42	< 0.01	5.44 ± 1.41	5.72 ± 1.42	< 0.01
BMI (kg/m^2^)	28.70 ± 5.90	27.52 ± 5.82	< 0.01	29.29 ± 6.31	27.45 ± 6.00	< 0.01
Ratio.of.family.income.to.poverty	2.70 ± 1.60	2.34 ± 1.50	< 0.01	2.76 ± 1.58	1.65 ± 1.26	< 0.01
Calcium	938.01 ± 589.10	835.48 ± 580.48	< 0.01	893.90 ± 524.96	985.56 ± 733.72	0.47
Alcohol	11.48 ± 28.94	13.09 ± 45.93	0.85	10.00 ± 26.08	29.86 ± 86.16	< 0.01
Fasting.glucose	6.07 ± 1.44	6.18 ± 1.54	0.02	6.39 ± 1.58	6.31 ± 1.52	0.21
HDL	52.86 ± 16.26	53.56 ± 16.28	0.15	48.88 ± 14.87	59.33 ± 17.06	< 0.01
Race			< 0.01			< 0.01
Mexican American (%)	2,759 (19.13%)	92 (7.56%)		634 (13.09%)	26 (9.70%)	
Other hispanic (%)	1,441 (9.99%)	144 (11.83%)		558 (11.52%)	21 (7.84%)	
Non-hispanic white (%)	6,432 (44.59%)	212 (17.42%)		1,738 (35.89%)	102 (38.06%)	
Non-hispanic black (%)	2,820 (19.55%)	435 (35.74%)		1,128 (23.29%)	110 (41.04%)	
Other race—including multi-racial (%)	973 (6.75%)	334 (27.44%)		785 (16.21%)	9 (3.36%)	
Education.level			< 0.01			< 0.01
Less than high school (%)	3,540 (24.54%)	354 (29.09%)		985 (20.34%)	98 (36.57%)	
High school or equivalent (%)	3,313 (22.97%)	314 (25.80%)		1,119 (23.11%)	90 (33.58%)	
College or above (%)	7,558 (52.40%)	548 (45.03%)		2,734 (56.45%)	79 (29.48%)	
Not recorded (%)	14 (0.10%)	1 (0.08%)		5 (0.10%)	1 (0.37%)	
Diabetes			< 0.01			< 0.01
Yes (%)	1,643 (11.39%)	175 (14.38%)		862 (17.80%)	26 (9.70%)	
No (%)	12,459 (86.37%)	998 (82.00%)		3,795 (78.36%)	237 (88.43%)	
Not recorded (%)	323 (2.24%)	44 (3.62%)		186 (3.84%)	5 (1.87%)	
Smoked			< 0.01			< 0.01
Yes (%)	6,696 (46.42%)	627 (51.52%)		2,201 (45.45%)	231 (86.19%)	
No (%)	7,724 (53.55%)	590 (48.48%)		2,641 (54.53%)	37 (13.81%)	
Not recorded (%)	5 (0.03%)	0 (0.00%)		1 (0.02%)	0 (0.00%)	

Data are presented as mean ± SD or n (%).

### Multiple regression analysis results

Hepatitis B-related respondents included in the population ended up with a total of 15,642 respondents, of which 1,217 have HBsAg (+) vs. 14,425 have HBsAg (–). According to the results of the multiple regression equation, we can see a significant difference in total femur BMD between HBsAg (+) and HBsAg (–) respondents: −0.022 (−0.031, −0.013) (*p* < 0.01). In Model 2, which was adjusted for age, gender, and ethnicity of respondents, there was also a significant difference in total femoral BMD between HBsAg (+) and HBsAg (–) respondents: −0.018 (−0.026, −0.009) (*P* < 0.01). In Model 3, adjusted for all covariates, there was no significant difference in total femur BMD between HBsAg (+) and HBsA (–) subjects though −0.002 (−0.010, 0.005) (*P* = 0.51). However, total femur BMD was reduced in HBsAg (+) respondents compared to HBsAg (–) respondents in Model 3, but HBsAg (+) respondents had significantly lower total femur BMD than HBsAg (–) respondents (the results of this analysis are shown in Table 2).

**Table 2:** β (95% CIs) for decreased bone mineral density among respondents with BMD, according to HBsAg or HCV-RNA seropositivity.

	**Hepatitis B and C**					
	**HBsAg (–)**	**HBsAg (**+**)**	***P*** **-value**	**HCV-RNA (–)**	**HCV-RNA (**+**)**	***P*** **-value**
**TFB**						
Model 1 β (95% CI) *P*-value	0	−0.022 (−0.031, −0.013)	< 0.01	0	0.007 (−0.012, 0.025)	0.48
Model 2 β (95% CI) *P*-value	0	−0.018 (−0.026, −0.009)	< 0.01	0	−0.043 (−0.059, −0.026)	< 0.01
Model 3 β (95% CI) *P*-value	0	−0.002 (−0.010, 0.005)	0.51	0	−0.015 (−0.032, 0.002)	0.07
Model 4 β (95% CI) *P*-value	0	−0.013 (−0.021, −0.005)	< 0.01	0	−0.027 (−0.045, −0.009)	< 0.01
**FNB**						
Model 1 β (95% CI) *P*-value	0	−0.024 (−0.033, −0.015)	< 0.01	0	0.029 (0.011, 0.046)	< 0.01
Model 2 β (95% CI) *P*-value	0	−0.010 (−0.018, −0.002)	0.01	0	−0.021 (−0.037, −0.005)	0.01
Model 3 β (95% CI) *P*-value	0	0.002 (−0.005, 0.009)	0.62	0	0.000 (−0.016, 0.017)	0.96
Model 4 β (95% CI) *P*-value	0	−0.007 (−0.015, 0.000)	0.06	0	−0.009 (−0.027, 0.008)	0.29
**TSB**						
Model 1 β (95% CI) *P*-value	0	−0.023 (−0.033, −0.013)	< 0.01	0	0.016 (−0.008, 0.040)	0.19
Model 2 β (95% CI) *P*-value	0	−0.020 (−0.030, −0.010)	< 0.01	0	−0.035 (−0.058, −0.012)	< 0.01
Model 3 β (95% CI) *P*-value	0	−0.008 (−0.017, 0.001)	0.08	0	−0.014 (−0.040, 0.012)	0.28
Model 4 β (95% CI) *P*-value	0	−0.015 (−0.025, −0.006)	< 0.01	0	−0.019 (−0.046, 0.007)	0.15

TFB, total femur BMD; FNB, femoral neck BMD; TSB, total spinal BMD.

Model 1: Non-adjusted.

Model 2: Adjusted for age, gender, and race.

Model 3: Adjusted for age, gender, race, income level, education level, AST, ALT, BMI, smoking, alcohol consumption, calcium intake, HDL, uric acid, creatinine, blood calcium levels, blood glucose, and history of diabetes.

Model 4: Adjusted for age, gender, race, income level, education level, AST, ALT, smoking, alcohol consumption, calcium intake, HDL, uric acid, creatinine, blood calcium levels, blood glucose, and history of diabetes.

In the included population of HCV-RNA-associated respondents, there were 268 HCV-RNA (+) and 4,843 HCV-RNA (–) respondents. In the same way, we performed multiple regression analyses on these data. In Model 1, there was no significant difference in total femoral BMD between HCV-RNA (+) respondents and HCV-RNA (–) respondents 0.007 (−0.012, 0.025) (*p* = 0.48). In contrast, there was a significant difference in total femoral BMD between HCV-RNA (+) respondents and HCV-RNA (–) respondents in Model 2 −0.043 (−0.059, −0.026) (*P* < 0.01). In Model 3, there was no significant difference in total femur BMD between HCV-RNA (+) and HCV-RNA (–) respondents −0.015 (−0.032, 0.002) (*P* = 0.07), but HCV-RNA (+) respondents showed a significant reduction in total femur BMD. HCV-RNA (+) may also increase the risk of bone loss (the results of this analysis are shown in Table 2).

In Model 1 of the multiple regression equation for femoral neck BMD in HBsAg (+) vs. HBsAg (–) respondents, we can see a significant difference in femoral neck BMD between HBsAg (+) and HBsAg (–) respondents −0.024 (−0.033, −0.015) (*p* < 0.01). In Model 2, there was also a significant difference in femoral neck BMD between HBsAg (+) and HBsAg (–) subjects −0.010 (−0.018, −0.002) (*P* = 0.01). In Model 3, adjusted for all covariates, there was no significant difference in femoral neck BMD between HBsAg (+) and HBsAg (–) respondents 0.002 (−0.005, 0.009) (*P* = 0.62). The BMD of the femoral neck was lower in HBsAg (+) respondents than in HBsAg (–) respondents (the results of the analysis are shown in Table 2).

In Model 1, there was a significant difference in femoral neck BMD between HCV-RNA (+) respondents and HCV-RNA (–) respondents 0.029 (0.011, 0.046) (*p* < 0.01). However, the femoral neck BMD was to be increased in HCV-RNA (+) respondents. In Model 2, there was no significant difference in femoral neck BMD between HCV-RNA (+) respondents and HCV-RNA (–) respondents −0.021 (−0.037, −0.005) (*P* = 0.01), and there was also no significant difference in femoral neck BMD between HCV-RNA (+) and HCV-RNA (–) respondents in Model 3, 0.000 (−0.016, 0.017) (*P* = 0.96). In terms of femoral neck BMD, HCV-RNA (+) respondents did not appear to receive a significant effect on femoral neck BMD (the specific results of the analysis are shown in Table 2).

In the Model 1 multiple regression analysis Modelof HBsAg and spinal BMD, there was a significant difference in spinal BMD between HBsAg (+) respondents and HBsAg (–) respondents −0.023 (−0.033, −0.013) (*P* < 0.01). In Model 2, there was also a significant difference in spinal BMD between HBsAg (+) respondents and HBsAg (–) respondents −0.020 (−0.030, −0.010) (*p* < 0.01). In Model 3, there was no statistically significant difference in spinal BMD between the two −0.008 (−0.017, 0.001) (*P* = 0.08), but there was a reduction in spinal BMD in HBsAg (+) respondents compared to HBsAg (–) respondents. This suggests that HBsAg (+) may reduce the spinal BMD of patients (the results of this analysis are shown in Table 2).

In Model 1, the spinal BMD between HCV-RNA (+) respondents and HCV-RNA (–) respondents was not significantly different by 0.016 (−0.008, 0.040) (*p* = 0.19). In Model 2, there was a significant difference in spinal BMD between HCV-RNA (+) respondents and HCV-RNA (–) respondents −0.035 (−0.058, −0.012) (*p* < 0.01). In Model 3, there was no significant difference in spinal BMD between HCV-RNA (+) and HCV-RNA (–) respondents −0.014 (−0.040, 0.012) (*P* = 0.28). However, some reduction in spinal BMD has also been seen in HCV-RNA (+) respondents in Model 3 (the results of the analysis are shown in Table 2).

### Stratified analyses

We conducted separate stratified analyses for age and BMI. There were no significant differences in femoral and spinal BMD between respondents of different ages, regardless of positive or negative hepatitis B and C test results (Table 3). In multiple regression analyses with BMI groupings, there was also no significant difference in femoral and spinal BMD between respondents (Table 4).

**Table 3:** Stratified analyses of bone mineral density in respondents, according to age in HBsAg or HCV-RNA seropositivity.

	**Hepatitis B and C**					
	**HBsAg (–)**	**HBsAg (**+**)**	***P*** **-value**	**HCV-RNA (–)**	**HCV-RNA (**+**)**	***P*** **-value**
**TFB**						
20–39	0	−0.001 (−0.023, 0.021)	0.90	–	–	–
40–59	0	−0.006 (−0.017, 0.005)	0.28	0	−0.011 (−0.032, 0.010)	0.32
60–70	0	−0.009 (−0.021, 0.003)	0.16	0	−0.012 (−0.043, 0.020)	0.47
**FNB**						
20–39	0	0.002 (−0.020, 0.024)	0.87	0	−0.001 (−0.072, 0.071)	0.99
40–59	0	−0.001 (−0.011, 0.010)	0.87	0	0.011 (−0.010, 0.033)	0.30
60–70	0	−0.007 (−0.019, 0.005)	0.23		−0.015 (−0.046, 0.016)	0.35
**TSB**						
20–39	0	0.004 (−0.017, 0.025)	0.70	0	−0.025 (−0.109, 0.059)	0.56
40–59	0	−0.012 (−0.025, 0.001)	0.07	0	0.005 (−0.026, 0.036)	0.77
60–70	0	−0.020 (−0.037, −0.003)	0.02	0	−0.050 (−0.103, 0.004)	0.07

Adjusted for gender, race, income level, education level, AST, ALT, BMI, smoking, alcohol consumption, calcium intake, HDL, uric acid, creatinine, blood calcium levels, blood glucose, and history of diabetes.

**Table 4:** Stratified analyses of bone mineral density in respondents, according to BMI in HBsAg or HCV-RNA seropositivity.

	**Hepatitis B and C**					
	**HBsAg (–)**	**HBsAg (**+**)**	***P*** **-value**	**HCV-RNA (–)**	**HCV-RNA (**+**)**	***P*** **-value**
**TFB**						
< 18.5	0	−0.006 (−0.056, 0.044)	0.82	0	0.034 (−0.111, 0.180)	0.65
18.5–24.9	0	0.002 (−0.011, 0.015)	0.77	0	−0.022 (−0.051, 0.006)	0.13
24.9–30	0	0.000 (−0.012, 0.012)	0.98	0	−0.016 (−0.045, 0.012)	0.26
>30	0	−0.003 (−0.017, 0.011)	0.69	0	−0.015 (−0.049, 0.018)	0.37
**FNB**						
< 18.5	0	−0.002 (−0.050, 0.046)	0.94	0	0.000 (−0.141, 0.141)	0.99
18.5–24.9	0	0.003 (−0.010, 0.015)	0.67	0	−0.012 (−0.040, 0.016)	0.40
24.9–30	0	0.006 (−0.005, 0.017)	0.31	0	−0.007 (−0.034, 0.021)	0.63
>30	0	0.000 (−0.014, 0.015)	0.96	0	0.020 (−0.014, 0.055)	0.25
**TSB**						
< 18.5	0	−0.008 (−0.075, 0.058)	0.80	0	0.248 (0.004, 0.491)	0.06
18.5–24.9	0	−0.006 (−0.022, 0.009)	0.42	0	−0.010 (−0.057, 0.036)	0.67
24.9–30	0	−0.005 (−0.020, 0.010)	0.55	0	−0.018 (−0.060, 0.024)	0.41
>30	0	−0.012 (−0.030, 0.006)	0.19	0	−0.026 (−0.073, 0.020)	0.27

Adjusted for age, gender, race, income level, education level, AST, ALT, smoking, alcohol consumption, calcium intake, HDL, uric acid, creatinine, blood calcium levels, blood glucose, and history of diabetes.

### The detection of linear relationships

**Figure 3:**
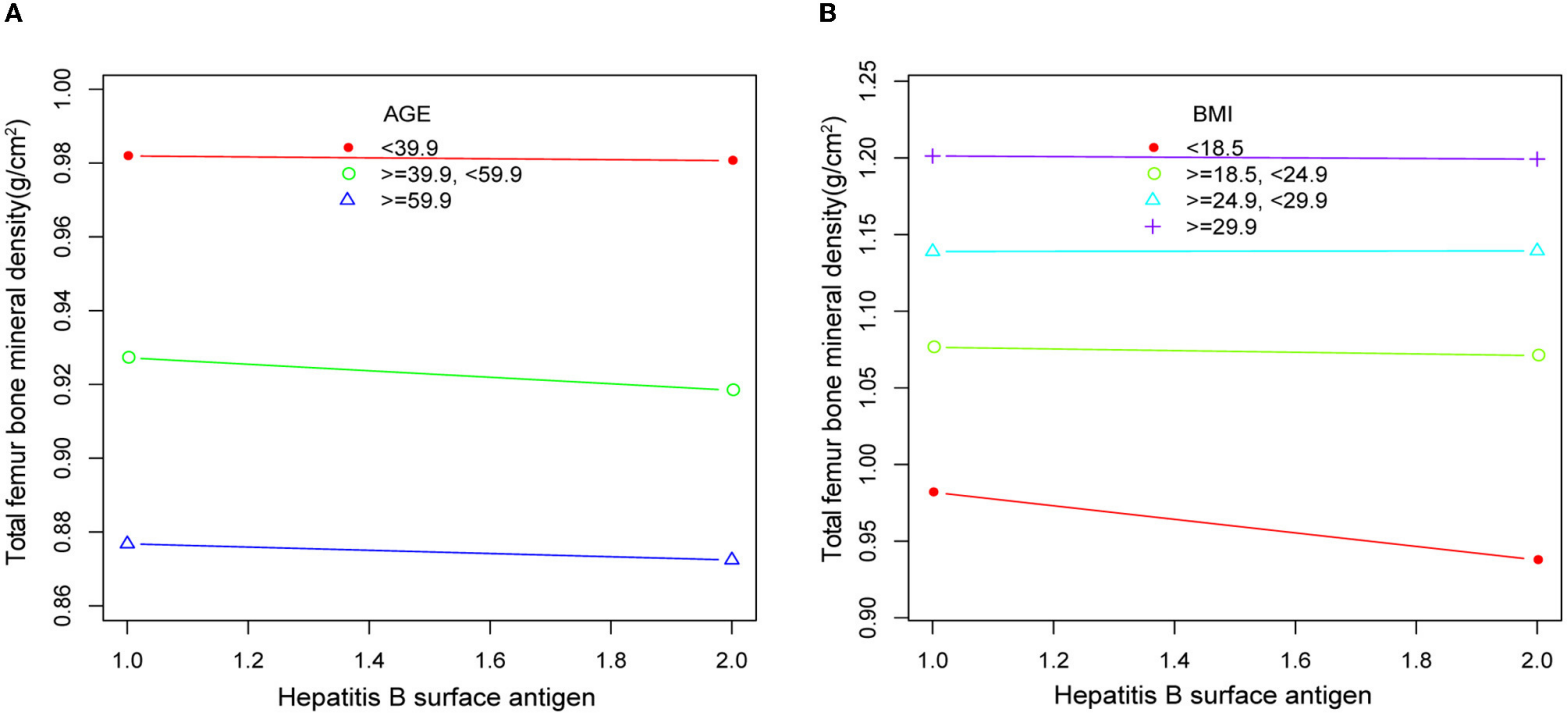
Smooth curve plots of bone mineral density in respondents according to age **(A)** and BMI **(B)** in HBsAg or HCV-RNA seropositivity. Adjusted for gender, race, income level, education level, AST, ALT, BMI, smoking, alcohol consumption, calcium intake, HDL, uric acid, creatinine, blood calcium levels, blood glucose, and history of diabetes.

Body mass index (BMI) was categorized as BMI ≤ 18.5, 18.5 < BMI ≤ 25, 25 < BMI ≤ 30, and BMI > 30. Age was categorized as age < 40 years, 40 ≤ age < 60 years, and age ≥ 60 years. All covariates were included, and a smooth curve was fitted. The resulting smooth curve plots showed little variation in total femoral BMD across the same age and BMI ranges (Figure 3).

## Discussion

Hepatitis, as a common infectious disease worldwide, is prone to liver cirrhosis and even liver cancer in its end stage (24, 25). Osteoporosis is a common complication in patients with hepatitis, and it is even more prevalent in patients with liver cirrhosis. However, there is still no definitive conclusion as to whether infection with the hepatitis virus directly causes a decrease in bone mass or even osteoporosis. Some studies had suggested that patients with hepatitis and liver cirrhosis are at higher risk of developing reduced bone BMD and osteoporosis (26, 27). Some studies had suggested that the long-term use of antiviral drugs in patients with hepatitis could lead to increased bone loss and impairment of renal function, which will lead to an increased risk of osteoporosis in patients with hepatitis (28, 29). However, studies on the relationship between viral hepatitis-related indicators and the decline in bone BMD are still scarce and have not been able to draw definitive conclusions. For these reasons, we collected the data related to hepatitis B surface antigen, hepatitis C RNA, femoral BMD, and spinal BMD from the NHANES database and perform a multiple regression analysis on these data. The aim of the study was to analyze whether positive serological indicators of viral hepatitis in US adults are associated with reduced BMD.

According to our final multiple regression analysis results, the BMD of HBsAg (+) and hepatitis C RNA (+) respondents was lower than serologically negative adults after combining various covariables that may affect the BMD of adults who were serologically negative, indicating that our final results are reliable. Furthermore, our smooth plots showed that the BMD of the hepatitis virus seropositive respondents was significantly lower than that of the hepatitis virus seronegative respondents, and these results largely validate the association between hepatitis virus infection and reduced BMD. Combined with the current research, bone loss in patients with hepatitis may be caused by the metabolism of the body and a series of inflammatory reactions after infection with the hepatitis virus. Because abnormal metabolism can easily lead to malnutrition, systemic inflammatory response and malnutrition can cause skeletal muscle loss, both of which are risk factors for bone loss (30–32). The interaction of various factors increases the risk of bone loss in patients with hepatitis virus infection.

In addition, the results of our analysis after removing the covariate BMI from Model 4 were statistically significant, with more significant differences in BMD between serologically positive and negative respondents. Furthermore, previous studies had suggested that BMI and age may be negatively correlated with BMD and that higher BMI and increasing age may lead to lower BMD in patients (33–36). Therefore, we conducted stratified analyses of the two covariates of BMI and age, with the aim of identifying the degree of influence of BMI and age on our analysis results. Regardless of age or BMI, the results of the stratified analysis indicated that there were no significant differences in BMD among respondents of different ages and BMIs. Moreover, the smooth curve plots of age and BMI also showed little variation in total femoral BMD across the same age and BMI ranges. Such results further validate the stability and accuracy of the results of our Model 4 analysis. We speculated that the infection with hepatitis B or C virus can cause an inflammatory response and metabolic disturbances in the body, leading to a decrease in BMD in the bones, which can increase the risk of osteoporosis.

Other studies had also suggested that hepatitis B and C virus serologically positive respondents show decreased BMD in the femur and spine compared to negative respondents, which may increase the risk of osteoporosis (37–39). In a study involving 51,144 respondents on the relationship between positive hepatitis B surface antigen and BMD in Taiwan (37), the results of their multiple regression analysis suggested a negative association between HBV positivity and BMD. HBV infection has a significant impact on the development of reduced BMD in the Taiwanese adult population. Similarly, the results from a national data study in Korea (38) suggested that serological positivity of hepatitis B is significantly associated with reduced BMD in men. In terms of hepatitis C-related studies, a meta-analysis (39) study suggested an increased risk of osteoporosis in patients with HCV infection. However, we did not believe that the results of these studies can be extrapolated to the BMD status of adults with viral hepatitis in the United States. This is because there are ethnic and lifestyle differences between countries, which may have different effects on bone mass. Therefore, our study can more truly reflect the BMD status of American adult patients with viral hepatitis.

Of course, there are some studies suggesting that the cause of osteoporosis in patients with hepatitis or liver cirrhosis is not caused by the infection with the hepatitis virus, but rather a decline in liver function that causes abnormal bone metabolism. Abnormal bone metabolism could lead to the deceased of bone synthesis and increased bone resorption, which would further lead to a decrease in BMD and osteoporosis. In a study of subjects from several hospitals in Taiwan (40), serum BAP and CTX levels were found to be higher in patients with chronic non-cirrhotic hepatitis C. These results implied that the early bone loss in patients with chronic non-sclerotic hepatitis C may be due to increased bone resorption. Several studies had also suggested that although the current mechanism of action between hepatitis virus infection of the liver and BMD is unclear, the physiological responses grown by various inflammatory factors following infection with the virus tend to stimulate osteoclast formation. Increased osteoclast formation could lead to a decrease in bone formation along with increased bone resorption, which could further lead to a decrease in BMD in patients with hepatitis (41–43).

Compared with some previous clinical studies, the samples of our study come from the NHANES database in the United States. Due to the relatively large sample size of these data, which is representative of the sample of adult respondents related to HBB and HBC in the United States, our research results are objective to a certain extent. Moreover, the proven sample follow-up of the NHANES database can provide a reliable basis for our analytical results. These are some of the advantages that we have in this study.

Of course, our current study also has certain limitations. First, this study is a cross-sectional observational study, and it can only analyze the relationship between hepatitis-related serological indicators and bone BMD. Second, the data included in this study do not include the specific medication status of patients infected with hepatitis B and C. For example, the use of tenofovir may increase the risk of reduced BMD. However, the NHANES database lacks information on tenofovir use in patients with hepatitis B. Therefore, a possible bias of tenofovir on the results of the analysis cannot be excluded. Third, glomerular filtration rates in chronic kidney disease and cirrhosis are also strongly associated with reduced BMD, and these data are not available in the NHANES database. We also cannot rule out the possibility that glomerular filtration rate and cirrhosis may bias the results of the study. Finally, as this is a large national survey, there may be some confounding factors due to measurement error and some unmeasured variables, and these potential confounding factors may have an impact on the results of our analysis.

## Conclusion

Following multiple regression analysis of hepatitis serologic indicators and BMD, we find that serologic HBsAg (+) and HCV-RNA (+) may be associated with an increased risk of reduced bone mass in patients. This suggests the importance of monitoring and preventing bone loss in our hepatitis serology-positive patients.

## Data availability statement

Publicly available datasets were analyzed in this study. This data can be found here: The datasets generated and analyzed in the current study are available at NHANES website: https://www.cdc.gov/nchs/nhanes/index.htm.

## Ethics statement

The protocols of NHANES were approved by the Institutional Review Board of the National Center for Health Statistics, CDC (https://www.cdc.gov/nchs/nhanes/irba98.htm). NHANES has obtained written informed consent from all participants. The patients/participants provided their written informed consent to participate in this study. Written informed consent was obtained from the individual(s) for the publication of any potentially identifiable images or data included in this article.

## Author contributions

TF conceived the theme and take responsibility for the article. JT and ZY are responsible for conceiving, writing the manuscript, and as well as analyzing the data. WH is responsible for the collation and collection of data and as well as the production of charts and graphs. All authors contributed to the article and approved the submitted version.
